# Homocysteinemia and early outcome of acute ischemic stroke in elderly patients

**DOI:** 10.1002/brb3.460

**Published:** 2016-04-05

**Authors:** Paola Forti, Fabiola Maioli, Giorgia Arnone, Maura Coveri, Gian Luca Pirazzoli, Marco Zoli, Gaetano Procaccianti

**Affiliations:** ^1^ Department of Medical and Surgical Sciences (DIMEC)University of BolognaBolognaItaly; ^2^ Geriatric Stroke UnitMedical DepartmentMaggiore HospitalBolognaItaly; ^3^ Neurology Stroke UnitInstitute of Neurological Sciences (IRCCS)Maggiore HospitalBolognaItaly

**Keywords:** Acute ischemic stroke, over 80 years oldstroke outcome, total homocysteine

## Abstract

**Objectives:**

Plasma total homocysteine (tHcy) is a risk factor for ischemic stroke (IS) but its relationship with IS outcome is uncertain. Moreover, previous studies underrepresented older IS patients, although risk of both hyperhomocysteinemia and IS increases with age. We investigated whether, in elderly patients with acute IS, tHcy measured on admission to the Stroke Unit (SU) is an independent predictor of SU discharge outcomes.

**Materials and Methods:**

Data are for 644 consecutive patients aged 80.3 ± 8.7 years, admitted to an Italian SU with diagnosis of acute IS. Plasma tHcy was measured on SU admission. Investigated outcomes included mortality during SU stay and poor functional status (modified Rankin Scale score ≥3) at SU discharge for survivors. The association of plasma tHcy with the study outcomes was assessed using Odds Ratios (OR) and their corresponding 95% confidence intervals (95%CI) from logistic regression models adjusted for demographics, pre‐stroke features, IS severity, and laboratory data on SU admission (serum C‐reactive protein, serum albumin, and renal function).

**Results:**

Median plasma tHcy was 16.7 *μ*mol/L (interquartile range, 13.0–23.3 *μ*mol/L). Outcome incidence was 5.3% for mortality and 49.7% for poor functional status. Plasma tHcy was unrelated to mortality in both univariate and multivariable‐adjusted analyses. Conversely, plasma tHcy was associated with poor functional status of survivors in univariate analyses (*P* = 0.014). Multivariable‐adjusted analyses showed that, compared to normal homocysteinemia (tHcy <16 *μ*mol/L), risk of being discharged with poor functional status significantly increased for moderate (tHcy ≥30 mol/L) but not mild (16.0–29.9 *μ*mol/L) hyperhomocysteinemia.

**Conclusions:**

In elderly patients with acute IS, high admission plasma tHcy is unrelated to mortality during SU stay but is an independent predictor of poor functional status at SU discharge in survivors. The association, however, is limited to patients with moderate hyperhomocysteinemia.

## Introduction

Blood total homocysteine (tHcy) is a marker of vitamin B deficiency and a nontraditional risk factor for acute ischemic stroke (IS) (Medina et al. 2001; Manolescu et al. 2010). Possible mechanisms include: endothelial toxicity, via both direct and indirect mechanisms (altered DNA methylation due to vitamin B deficiency, inflammation, and products from oxidative stress), proliferation of arterial smooth muscle, and coagulation promotion (Medina et al. 2001; Manolescu et al. 2010; Kalani et al. 2013).

High blood tHcy at IS onset might also predict worse clinical outcome in the early phase of IS because, in addition to damaging vascular walls, tHcy also has direct neurotoxic properties and affects regulation of the genes involved in brain response to ischemic injury (Medina et al. 2001; Manolescu et al. 2010; Kalani et al. 2013). However, previous cohort studies of the association between tHcy and early outcome of IS provided conflicting results (Perini et al. 2005; Haapaniemi et al. 2007; Yoldas et al. 2007; Song et al. 2010; Tu et al. 2013; Kwon et al. 2014; Saberi et al. 2014; Zhong et al. 2014). Moreover, all of these investigations underrepresented in over 80 year olds, although incidence of hyperhomocysteinemia, incidence of IS, and risk of poor IS outcome all increase with age (Medina et al. 2001; Chen et al. 2010).

This study investigated whether tHcy measured on admission to Stroke Unit (SU) is an independent predictor of SU discharge outcomes in a cohort of patients with acute IS including a substantial proportion of over 80 year olds.

## Methods

### Patients

Data for this study are from an ongoing database established at the Maggiore SU for collection of data about characteristics of older patients with acute IS (Forti et al. 2013). Between October 2007 and December 2013, prospective data were recorded for 1120 patients ≥60 year admitted to the SU of the Maggiore Hospital (Bologna, Italy) with diagnosis of acute IS based on clinical criteria (Hatano 1976) and brain CT‐scan performed within 24 h after hospital admission. Patients undergoing intravenous thrombolysis were not recorded in our database because, in Italy, this treatment is not yet licensed for age ≥80 year.

The Maggiore SU (10 beds Neurology SU, 10 beds Geriatric SU) admits about 90% of all stroke patients from a catchment area of about 220,000 inhabitants. About 98% of IS patients are directly referred from the Emergency Department, on average 12 h from arrival. Within 24 h after SU admission, all IS patients undergo a complete clinical and neurological examination, ECG, carotid Doppler ultrasonography, transthoracic echocardiography, 24‐h Holter ECG (if a cardioembolic etiology is suspected), and laboratory tests performed on fasting blood and urine samples collected on the first morning after SU admission (on average 24 h from hospital admission and 48 h from IS onset). The Maggiore SU physician team includes both neurologists and geriatricians. Patients are treated according to institutional guidelines for stroke care. Exclusion criteria for this study were: incomplete laboratory data; homocysteinuria (plasma tHcy >100 *μ*mol/L (Medina et al. 2001)); thyroid disease (history of thyroid disease, current assumption of thyroid drugs before IS onset, or abnormal serum thyreotropin and free thyroxin on SU admission); current pre‐stroke use of drugs known to affect plasma tHcy (immunosuppressor, anticancer, or anticonvulsivant drugs); and severe renal dysfunction (estimated glomerular filtration [eGFR] rate <30 mL/min on SU admission).

### Ethical standard

The Maggiore Hospital Ethics Committee provided ethical approval; all patients (or their representatives) provided written informed consent.

### Covariates

The following information was derived from the database: demographic data (age and sex), pre‐stroke features, IS characteristics, and laboratory data on SU admission. Pre‐stroke features included: hypertension, defined as history of blood pressure >140/90 mmHg or current treatment; diabetes mellitus, defined as history of fasting glycemia >126 mg/dL in ≥2 measurements or current treatment (American Diabetes Association, 2013); atrial fibrillation, defined as history of chronic or paroxysmal atrial fibrillation or positive ECG during SU stay; hyperlipidemia, defined as admission serum lipids above standard reference values (total cholesterol ≥ 6.2 mmol/L, LDL‐cholesterol ≥ 4.1 mmol/L, or tryglicerides ≥ 1.70 mmol/L (Grundy et al. 2004)) or pre‐stroke use of lipid‐lowering drugs); comorbidity measured with the Charlson Comorbidity Index (CCI) (Charlson et al. 1987); and pre‐stroke‐modified Rankin Score (mRS) scale (Van Swieten et al. 1988) according to information provided by the patients and/or their primary caregiver. IS characteristics included: etiology according to the Trial of Org 10172 in Acute Stroke Treatment (TOAST) classification (Adams et al. 1993); and neurological severity according to the National Institutes of Health Stroke Scale (NIHSS) score (Brott et al. 1989) as assessed on SU admission. Plasma tHcy was measured using fluorescence polarization immunoassay (IMx analyzer, Abbott Laboratories, Abbott Park, IL). Serum levels of high‐sensitivity C‐reactive protein (CRP), creatinine, and albumin were measured by standard automatised methods. The eGFR rate was calculated from serum creatinine using the abbreviated Modification of Diet in Renal Disease formula (Froissart et al. 2005).

### SU discharge outcomes

Two separate outcomes were investigated: (1) mortality during SU stay for all patients; (2) poor functional status at SU discharge for patients discharged alive. Poor functional status was defined as mRS ≥3 at SU discharge for survivors with pre‐stroke mRS <3 or any increase in mRS at SU discharge for survivors with pre‐stroke mRS ≥3 (Weisscher et al. 2008).

### Statistics

Continuous variables were reported as mean ± SD except for NIHSS, tHcy, and CRP that, because of their skewed distribution, were reported as median [interquartile range (IQR)]. Categorical variables were reported as frequencies (percentage). Student t‐test and *χ* ^2^‐test were used for univariate comparisons. For skewed variables, univariate analyses were performed after square‐root (NIHSS) or natural log‐transformation (CCI, tHcy, and CRP). The association of tHcy (treated as a continuous log‐transformed variable) with TOAST etiology and NIHSS score (categorized according to previously suggested cutoffs (Fonarow et al. 2012): 0–7,8–13, 14–21, ≥22) was investigated using ANOVA. The association of tHcy with the study outcomes was estimated using odds ratios (ORs) and their 95% confidence intervals (95% CI) from logistic regression models adjusted for age, sex, hypertension, diabetes mellitus, atrial fibrillation, hyperlipidemia, NIHSS, CRP, albumin, and eGFR. In preliminary analyses, further adjustment for TOAST etiology caused collinearity because of its correlation with NIHSS and did not significantly change results of logistic regression models. Therefore, TOAST etiology was not included as a covariate in the final logistic models. In logistic regression analyses, plasma tHcy was analyzed both as a continuous (log‐transformed) and a categorical variable in order to detect nonlinearly shaped associations with the study outcomes. Previously published cutpoints were used to define normal homocysteinemia (plasma tHcy <16 *μ*mol/L) and mild (16.0–29.9 *μ*mol/L) and moderate (≥30.0 *μ*mol/L) hyperhomocysteinemia (Medina et al. 2001).

All analyses were 2‐sided, conducted at a 0.05 level of significance, and performed using SPSS version 21.0 for Windows (IBM corp. Armonk, NY).

## Results

**Figure 1:**
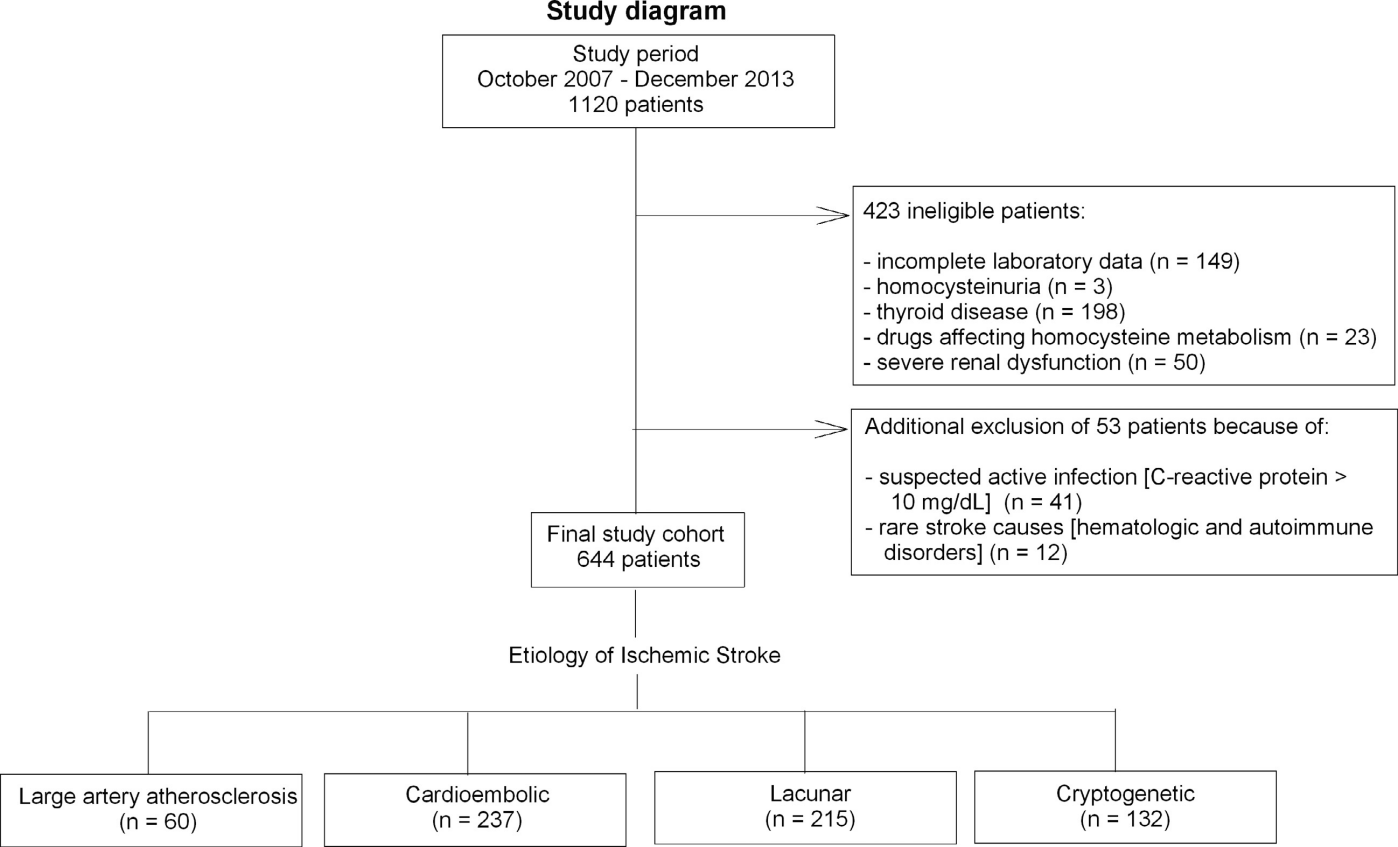
Study diagram.

After application of exclusion criteria and additional exclusion of patients with suspected active infection (CRP ≥ 10 mg/dL) or rare stroke causes (Fig. 1), the final study cohort included 644 patients. Mean age was 80.3 ± 8.7 years and about 51.2% of patients were women. Median length of stay in SU was 10 days (range 1–48). Excluded patients did not differ by demographics or length of stay (data not shown).

Median plasma tHcy of the study participants was 16.7 *μ*mol/L [13.0–23.3 *μ*mol/L] and above the reference level for hyperhomocysteinemia. When grouping the study patients by TOAST etiology, median tHcy was 17.5 *μ*mol/L [14.1–25.6 *μ*mol/L] for large‐artery atherosclerosis, 16.9 *μ*mol/L [13.6–23.4 *μ*mol/L] for cardioembolic, 17.4 *μ*mol/L [12.7–23.6 *μ*mol/L] for lacunar, and 15.2 *μ*mol/L [11.9–21.3 *μ*mol/L] for cryptogenetic etiology. In univariate ANOVA, tHcy was significantly different across TOAST etiology (*P* = 0.043). However, the association weakened below significance (*P* = 0.073) after controlling for possible confounders (age, sex, hypertension, diabetes mellitus, atrial fibrillation, hyperlipidemia, CCI, NIHSS, CRP, albumin, and eGFR). When grouping the study patients by categories of admission NIHSS score, median tHcy was 16.4 *μ*mol/L [12.7–22.5 *μ*mol/L] for score 0–7 (*n* = 399), 17.1 *μ*mol/L [13.4–23.6 *μ*mol/L] for score 8–13 (*n* = 98), 16.4 *μ*mol/L [12.0–26.0 *μ*mol/L] for score 14–21 (*n* = 90), and 17.7 *μ*mol/L [13.4–23.6 *μ*mol/L] for score ≥22 (*n* = 57). In univariate ANOVA, log‐transformed tHcy did not differ across NIHSS score categories (*P* = 0.641) and results did not change after controlling for TOAST etiology and the other confounders listed above (*P* = 0.774).

A total of 34 patients (5.3%) died during SU stay and, out of those discharged alive, 303 (49.7%) had poor functional status. Length of stay was shorter for patients who died during SU stay (8 [4–12] days) compared to survivors (10 [7–14] days, Mann–Whitney Test *P* = 0.012).

Univariate characteristics of patients grouped by the study outcomes are detailed in Table 1. Patients with unfavorable discharge outcomes were generally older, more likely to have atrial fibrillation, and more likely to have higher NIHSS and serum CRP on admission. Patients who died during SU stay were further characterized by lower serum albumin compared to those discharged alive. Among survivors, those with poor functional outcome at SU discharge were more likely to be females, to have hypertension, and to have lower eGFR rates compared to patients with good functional outcome. Univariate analyses also showed that plasma tHcy did not differ between those who died during SU stay and those discharged alive, but was significantly higher for survivors discharged from SU with poor functional status compared to survivors discharged with good functional status. Multivariable‐adjusted analyses (Table 2) confirmed that plasma tHcy, both as a continuous and a categorical variable, was unrelated to mortality during SU stay. Multivariable adjustment also weakened below statistical significance the association of functional status of survivors with plasma tHcy treated as a continuous variable. However, in multivariable‐adjusted analyses of plasma tHcy considered as a categorical variable, the odds of poor functional status at SU discharge doubled in survivors with moderate hyperhomocysteinemia compared to survivors with normal homocysteinemia. No similar association was found for survivors with mild hyperhomocysteinemia.

**Table 1:** Characteristics of the study patients by discharge outcome from Stroke Unit

Variables	Alive at discharge *n* = 610	Deaths *n* = 34	*P* value	Survivors with good functional outcome *n* = 307	Survivors with poor functional outcome *n* = 303	*P* value
Age, year	80.0 ± 8.7	84.0 ± 6.8	<0.001	77.0 ± 8.7	82.2 ± 8.1	<0.001
Male	300 (49.2)	14 (41.2)	0.364	169 (55.0)	131 (43.2)	0.004
Pre‐stroke mRS ≥3	105 (17.2)	10 (29.4)	0.071	49 (16.0)	56 (18.5)	0.410
Hypertension	477 (78.2)	28 (82.4)	0.566	228 (74.3)	249 (82.2)	0.018
Diabetes mellitus	198 (32.5)	10 (29.4)	0.712	98 (31.9)	100 (33.0)	0.775
Atrial fibrillation	137 (22.5)	20 (58.8)	<0.001	47 (15.3)	90 (29.7)	<0.001
Hyperlipidemia	238 (39.0)	9 (26.5)	0.143	120 (39.1)	118 (38.9)	0.971
CCI	1 [0–2]	2 [1–3]	0.144	1 [0–2]	1 [0–3]	0.698
Admission NIHSS	5 [2–11]	21 [17–26]	<0.001	3 [1–5]	9 [4–17]	<0.001
Plasma tHcy, *μ*mol/L	16.7 [13.0–23.3]	17.3 [11.6–23.5]	0.983	16.0 [12.6–21.9]	17.5 [13.6–24.7]	0.017
Serum CRP, mg/dL	0.56 [0.25–1.75]	2.55 [1.21–5.31]	<0.001	0.39 [0.18–1.08]	0.90 [0.34–2.41]	<0.001
Serum albumin, g/dL	3.5 ± 0.4	3.3 ± 0.5	0.016	3.5 ± 0.4	3.5 ± 0.4	0.946
eGFR, mL/min/1.73 m^2^	73.0 ± 21.7	70.8 ± 29.5	0.659	75.0 ± 21.0	71.1 ± 22.2	0.025

CCI, Charlson Comorbidity Index; CRP, C‐reactive protein; eGFR, estimated glomerular filtration rate; mRS, modified Rankin scale; NIHSS, National Institute of Health Stroke Scale; tHcy, total homocysteine. Continuous variables were reported as mean ± SD except for NIHSS, tHcy, and CRP that, due to their skewed distribution, were reported as median [interquartile range]. Categorical variables were reported as number of patients (%). *P*‐values are from Student *t*‐test (continuous variables) or *χ* ^2^‐test (categorical variables); for continuous variables with skewed distribution, Student t‐test was performed after square‐root (NIHSS) or natural log‐transformation (tHcy and CRP).

**Table 2:** Multivariable‐adjusted associations of admission plasma tHcy with discharge outcomes from Stroke Unit in elderly patients with acute ischemic stroke

Variables	Death during SU stay					Poor functional status				
	*n*	*N*	%	OR (95% CI)	*P* value	*n*	*N*	%	OR (95% CI)	*P* value
5 *μ*mol/L increase in plasma tHcy	34	644	5.3	1.02 (0.86–1.23)	0.770	303	610	49.7	1.09 (0.99–1.21)	0.072
Hyperhomocysteinemia										
Absent (<16 *μ*mol/L)	15	292	5.1	1.00		123	277	44.4	1.00	
Mild (16–29.9 *μ*mol/L)	14	274	5.1	0.63 (0.26–1.57)	0.323	137	260	52.7	1.44 (0.93–2.24)	0.105
Moderate (≥30 *μ*mol/L)	5	78	6.4	1.04 (0.30–3.58)	0.949	43	73	58.9	2.05 (1.05–4.01)	0.035

*n*, number of cases; *N*, number of exposed; %, percentage of cases; SU, Stroke Unit; tHcy, total homocysteine. Odds Ratios (OR) and their 95% confidence intervals (95% CI) are from logistic models adjusted for age, sex, hypertension, diabetes mellitus, atrial fibrillation, hyperlipidemia, Charlson Comorbidity Index, admission National Institutes of Health Stroke Scale score, and admission C‐reactive protein, albumin, and estimated glomerular filtration rate. Analyses for poor functional status were limited to patients discharged alive.

## Discussion

This study found that, in elderly patients with acute IS admitted to SU, high admission plasma tHcy was unrelated to mortality during SU stay but associated with poor functional status at SU discharge for those discharged alive. This association, however, was significant only for plasma tHct in the range for moderate hyperhomocysteinemia.

Findings from previous cohort studies of admission tHcy and early outcome of IS inpatients are very conflicting: some studies reported an association (Tu et al. 2013; Kwon et al. 2014; Zhong et al. 2014), but others did not (Perini et al. 2005; Haapaniemi et al. 2007; Yoldas et al. 2007; Song et al. 2010). One study even reported that high tHcy was associated with better outcome (Saberi et al. 2014). However, all of these cohorts underrepresented elderly subjects, particularly in over 80 year olds, and used a combined stroke outcome including both mortality and poor functional status. Other limitations include: small samples (Haapaniemi et al. 2007; Yoldas et al. 2007; Song et al. 2010; Tu et al. 2013; Kwon et al. 2014; Saberi et al. 2014); derivation from clinical trials (Kwon et al. 2014) or cohorts not receiving organized SU care (Haapaniemi et al. 2007; Tu et al. 2013; Zhong et al. 2014); possible confounding from IS severity or renal dysfunction (Perini et al. 2005; Tu et al. 2013; Kwon et al. 2014; Saberi et al. 2014; Zhong et al. 2014); and lack of generalizability to non‐East Asian patients (Song et al. 2010; Tu et al. 2013; Kwon et al. 2014; Zhong et al. 2014).

Studies of admission tHcy and long‐term outcome of IS after hospital discharge in adults also produced conflicting results, with some studies finding an association (Pniewski et al. 2003; Wu et al. 2013), whereas others did not (Okubadejo et al. 2008). Sample sizes, however, were very small and, again, IS outcome was defined as combination of death and poor functional status.

Our study contributes to explain the heterogeneity of these previous studies because it shows that, in elderly IS patients, the association of admission tHcy with SU discharge outcomes is limited to poor functional status of patients discharge alive and has a threshold response, with risk doubling for tHcy above the cutoff for moderate hyperhomocysteinemia but exhibiting no variation for tHcy in the range from normal to the upper limit of mild hyperhomocysteinemia.

These findings are consistent with the hypothesis that, in IS patients, circulating tHcy may be an indicator of postacute vascular damage and neurotoxic effects that aggravate the original neuronal injury and interfere with the reparation processes. Possible tHcy‐related mechanisms underlying these adverse effects include: impaired adhesion and proliferation of endothelium with further disruption of the blood–brain barrier; promotion of coagulation; inhibition of membrane metabolism in neuronal cells; induction of neuronal excitotoxicity; and transformation of tHcy into highly reactive compounds that may trigger the inflammatory cascade and the production of free radicals (Medina et al. 2001; Manolescu et al. 2010). Moreover, tHcy itself or key enzymes of its metabolism may affect genes involved in the regulation of neuronal responses to ischemia, including apoptosis, and the modulation of reparative processes in the ischemic brain (Kalani et al. 2013). The lack of prognostic significance of high admission tHcy with vital status at SU discharge may be explained by the fact that, for mortality measured over a very short time interval such as SU stay, neurological severity usually outclasses any other prognostic determinant of IS mortality (Silver et al. 1984; Koennecke et al. 2011). Therefore, the contribution of hyperphomocysteinemia to neurological damage might be observed only in patients who survive the most acute phase of ischemia. The threshold‐response shape of the association supports the hypothesis of hyperhmocysteinemia as an indicator of poststroke mechanisms, because it suggests that only very high tHcy levels are associated with metabolic processes significantly contributing to ischemic injury.

An alternative hypothesis is that admission tHcy is not related to IS functional outcome through poststroke processes but is an indicator of poor pre‐stroke health. According to this hypothesis, two explanatory mechanisms can be hypothesized. A first possibility is that tHcy acts as a marker of pre‐existing vascular diseases in their turn associated with greater brain ischemic damage (e.g. cardioembolic conditions). Indeed, hyperhomocysteinemia has acknowledged associations with thromboembolism and vascular diseases (Medina et al. 2001; Manolescu et al. 2010; Kalani et al. 2013). The Leiden‐85‐plus Study even showed that a single tHcy measurement, but not traditional vascular risk factors, predicted cardiovascular mortality in persons aged 85 year with no history of cardiovascular disease (De Ruijter et al. 2009). In our study, however, the association between admission tHcy and poor outcome was independent of TOAST etiology as well as of age, NIHSS, and several traditional vascular risk factors including CRP. The lack of statistically significant associations between tHcy and TOAST etiology found in our this study is consistent with available evidence that, due to its associations with both atherogenetic and cardioembolic processes, high tHcy is not the distinguishing feature of a unique IS subtype (Wiseman et al. 2014). The lack of association between admission tHcy and IS severity as measured by NIHSS further support the view that, in our study, circulating tHcy did not act as a proxy measurement for the primary causative agent of brain ischemia. A second, not mutually exclusive, possibility is that high tHcy might act as an aspecific marker of nonvascular pre‐existing chronic conditions that can have adverse effects on stroke outcome by increasing risk of poststroke complications and interfering with rehabilitation. Indeed, hyperhomocysteinemia is a consistent features of many nonvascular chronic conditions such as alterations of skeletal maintenance and neurodegenerative disorders (Schalinske and Smazal 2012). In community‐dwellers, hyperhomocysteinemia also predicts noncardiac mortality (Vollset et al. 2001; Bates et al. 2010). It remains unclear, however, whether high tHcy is a causative factor or merely an epiphenomenon of disease. Although in vitro and in vivo evidence supports a causative role of hyperhomocysteinemia in several pathogenetic processes, the effectiveness of tHcy‐lowering therapy for prevention of human disease and mortality remains unproven (Medina et al. 2001; Manolescu et al. 2010; Schalinske and Smazal 2012; Kalani et al. 2013). We cannot entirely discard the hypothesis that, in our study, hyperhomocysteinemia just reflected some pre‐stroke nonvascular chronic condition. However, our logistic models took into account not only several individual pre‐stroke chronic conditions but also the burden of pre‐stroke comorbidity as measured using the Charlson Comorbidity Index.

Finally, a recent investigation of 214 Chinese patients with acute stroke reported an independent association of admission tHcy with poststroke fatigue (Wu et al. 2015), a condition that may hamper rehabilitation. The study, however, had a case–control, cross‐sectional design and further data are needed to ascertain this association.

Our findings are relevant for two reasons. First, by showing a threshold‐dependent association of admission tHcy with early functional status but not mortality of IS patients, this study contributes to explain the inconsistent results of previous investigations of this issue and provide information about possible sources of confounding for future clinical trials in stroke patients including tHcy measurement. Second, our findings suggest that, although statistically associated with SU discharge outcome, admission tHcy may not be actually clinically useful for SU physicians because the association becomes relevant only for a minority of patients. Although more than a half of our patients had tHcy above the normal range, only 10% of survivors were classified as having moderate hyperhomocysteinemia. Therefore, routine measurement of plasma tHcy on admission of elderly IS patients may not be cost‐effective.

Strengths of our study include: use of data from a Caucasian SU cohort with a large number of over 80 year olds, measurement of plasma tHcy within 48 hours from IS onset, and adjustment for several major confounders. Our study also has limitations. First, the study design cannot establish a causal relationship between tHcy and poor functional outcome. Second frailty, vitamin B status, and pre‐stroke use of B‐vitamin supplementations were not measured. Third, adjustment for NIHSS score might have been insufficient to fully control for IS severity. Fourth, although we knew length of stay in SU for all the study participants, we chose to not use Cox regression analysis to investigate the association of admission tHcy with the study outcomes. This decision was based on our inability to take into account the confounding effect of factors that, although not strictly related to IS, can all the same affect SU length of stay (e.g. occurrence of medical complications, patient/family's request for transfer to another hospital nearer to their home, variations in SU discharge planning related to seasonal and occasional overcrowding). Finally, our findings may not apply to other SU settings and to postdischarge stroke outcomes. SU outcomes are used as quality indicators of acute treatment and are important for health costs, hospital discharge planning, and resource allocation for postacute care services. However, assessment of outcome at SU discharge rather than at a fixed time interval might introduce biases related to local peculiarities in organization of discharge pathways.

In conclusion, this study shows that in elderly patients with acute IS, admission plasma tHcy is an independent predictors of functional status at SU discharge in those discharged alive, but limited to plasma tHcy in the range for moderate hyperhomocysteinemia. This threshold‐response and the lack of association with mortality during SU stay may limit the clinical usefulness of admission tHcy as a prognostic marker of early IS outcome in elderly persons.

## Conflict of Interest

The authors declare that they have no conflict of interest.
